# Sacro-Iliite à *Erysipelothrix Rhusiopathiae* révélant une endocardite tricuspide, premier cas rapporté sur le bouclier des Guyanes : cas clinique et revue de la littérature

**DOI:** 10.48327/mtsi.v2i3.2022.256

**Published:** 2022-07-27

**Authors:** Mathilde KHENG, Jean Francky ALEXIS, Gaëlle WALTER, Émilie MOSNIER, Thomas MALMONTET, Magalie PIERRE DEMAR, Vilyn TAUCH, Guillaume VESIN, Philippe ABBOUD, François ROQUES, Félix DJOSSOU, Loïc EPELBOIN

**Affiliations:** 1 Unité des maladies infectieuses et tropicales, Centre hospitalier Andrée Rosemon, Cayenne, Guyane, France; 2 Service de cardiologie, Centre hospitalier Andrée Rosemon, Cayenne, Guyane, France; 3 Laboratoire de microbiologie, Centre hospitalier Andrée Rosemon, Cayenne, Guyane, France; 4 Service de radiologie, Centre hospitalier Andrée Rosemon, Cayenne, Guyane, France; 5 Service de chirurgie thoracique, Centre hospitalier universitaire de Martinique, Fort-de-France, Martinique, France

**Keywords:** *Erysipelothrix rhusiopathiae*, Sacro-iliite, Endocardite infectieuse, Infection ostéoarticulaire, Zoonose, Hôpital, Cayenne, Guyane, *Erysipelothrix rhusiopathiae*, Sacroiliitis, Infective endocarditis, Osteoarticular infection, Zoonosis, Hospital, Cayenne, French Guyana

## Abstract

Nous rapportons le cas d'une patiente de 53 ans, sans antécédent, dont le bilan étiologique d'une douleur de hanche fébrile, réalisé à l'hôpital de Cayenne, a révélé une sacro-iliite droite associée à une endocardite infectieuse tricuspide à *Erysipelothrix rhusiopathiae*. Nous présentons une revue de la littérature des infections ostéo-articulaires à *E. rhusiopathiae*. Aucun cas d'infection ostéo-articulaire à *E. rhusiopathiae* n'avait encore été décrit en Amérique latine.

## Abstract (en)

We report here an atypical case of acute sacroiliitis caused by *Erysipelothrix rhusiopathiae* revealing tricuspid endocarditis in a 53-year-old woman without medical history. She was admitted to Cayenne hospital because of intense right hip and thigh pain, associated with fever. A right sacroiliitis was visible on the computed tomography (CT) scan, confirmed on MRI. Transesophageal echocardiography revealed a large mobile tricuspid vegetation. Blood cultures were positive for *E. rhusiopathiae*. CT scan showed pulmonary alveolar opacities, consistent with septic emboli. Clinical improvement was obtained under ceftriaxone followed by ciprofloxacin for 6 weeks of treatment. We present a review of bone and joint infections caused by *E. rhusiopathiae*. So far, not a single case has been reported in Latin America.

## Introduction

*E. rhusiopathiae* est un bacille Gram positif de distribution ubiquitaire. Son réservoir est animal: les porcs, bovins, volailles, poissons et crustacés sont ses principaux hôtes. Il peut persister jusqu’à plusieurs mois dans leur environnement. S'il peut faire partie de la flore commensale animale, il est toujours pathogène pour l'homme [27]. Les humains se contaminent par contact avec les animaux ou leur environnement. Une exposition professionnelle est retrouvée dans la plupart des cas [4]. D'autres facteurs de risque ont été rapportés: diabète, immunodépression, maladies inflammatoires chroniques, pathologie rénale ou hépatique chronique [11, 18, 31]. Trois entités cliniques ont été décrites chez l'homme: une forme cutanée localisée, appelée « rouget du porc », une forme cutanée diffuse et une forme systémique, avec bactériémie et/ou infection de sites stériles [27]. En cas de bactériémie, une endocardite infectieuse est fréquemment retrouvée [14, 26]. Les localisations ostéoarticulaires sont cependant inhabituelles. Nous rapportons ici un cas d'endocardite infectieuse à *E. rhusiopathiae*, révélée par une sacro-iliite, et présentons une revue des cas d'infection ostéo-articulaire à *E. rhusiopathiae* décrits dans la littérature.

## Cas Clinique

**Figure 1:**
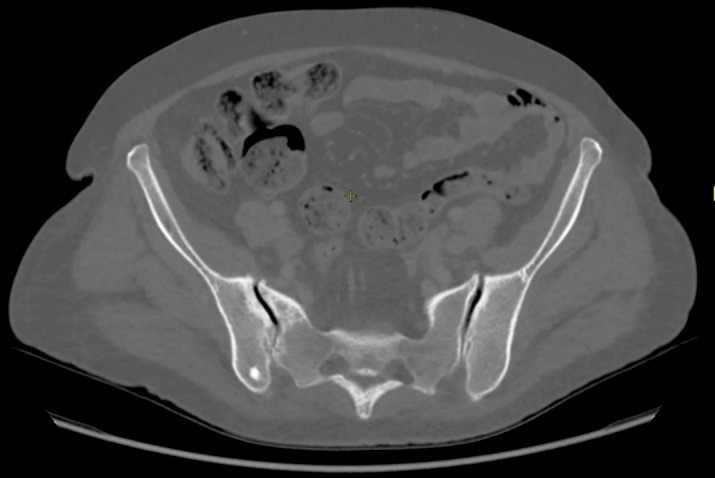
Sacro-iliite droite. Sclérose sous-chondrale et erosions sur le côté iliaque et sacré de l'articulation. Ostéophytes marginaux. Scanner Right sacroiliitis. Subchondral sclerosis and erosions on the iliac and sacral side of the joint. Marginal osteophytes. CT Scan

**Figure 2:**
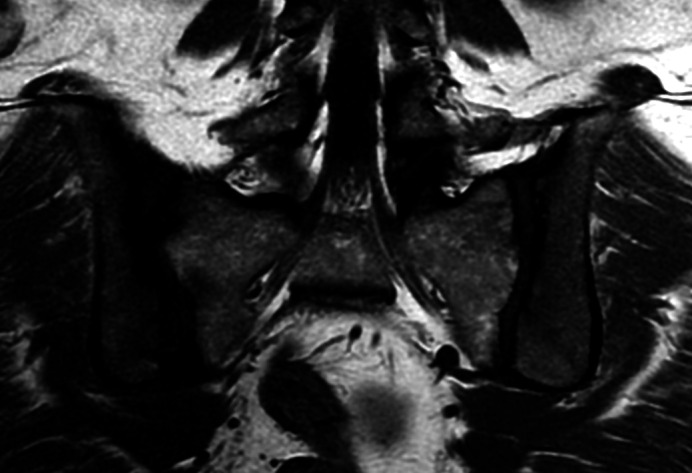
Sacro-iliite droite. Sclérose sous-chondrale (bande hypointense). IRM, image pondérée en T1 Right sacroiliitis. Subchondral sclerosis (hypointense band). MRI T1-weightened image

Une patiente de 53 ans, originaire de Guyane française, femme au foyer, sans antécédent notable, notamment d'immunodépression ou de comorbidité, consulte à l'hôpital de Cayenne pour une douleur de hanche droite d'horaire inflammatoire apparue 4 jours plus tôt, associée à de la fièvre. À l'examen clinique, la température est de 38,1 °C, sa hanche est chaude, douloureuse avec une amplitude limitée. À l'auscultation, un souffle aortique non connu est perçu. Au bilan biologique, il est constaté un syndrome inflammatoire biologique (protéine C-réactive à 119 mg/l) sans hyperleucocytose. Une coxarthrose bilatérale et une sacroiliite droite sont décrites au scanner (Fig. 1). L’échocardiographie transthoracique met en évidence une insuffisance mitrale, aortique et tricuspide avec remaniements au niveau de la valve tricuspide, évoquant une cardiopathie rhumatismale. Quelques jours après l'admission, deux hémocultures reviennent positives à *E. rhusiopathiae*. La souche est sensible aux céphalosporines, aux quinolones et aux cyclines, de sensibilité intermédiaire à la pénicilline et résistante aux aminoglycosides, à la vancomycine et à la rifampicine (Tableau I). La sensibilité aux macrolides/lincosamides n'a pas été étudiée, et aucun résultat de concentration moyenne inhibitrice n'est disponible. En reprenant l'interrogatoire, la patiente rapporte une coupure en préparant du poisson deux mois auparavant. Au moment de l'hospitalisation, l'identification du poisson n'avait pas été précisée. L'IRM pelvienne confirme la sacroiliite droite (Fig. 2). L’échocardiographie trans-oesophagienne retrouve une végétation tricuspide mobile, d'une taille de 8 × 13 mm. Le scanner cérébral et thoracoabdominopelvien met en évidence des opacités alvéolaires pulmonaires bilatérales, compatibles avec des emboles septiques, sans autre localisation secondaire. Le diagnostic d'endocardite tricuspide infectieuse à *E. rhusiopathiae* compliquée d'emboles septiques pulmonaires, révélée par une sacro-iliite droite, est retenu. Un traitement par ceftriaxone 4 grammes par jour est introduit. Le lavage chirurgical n'est pas des lésions pulmonaires. Aucune rechute infectieuse ou complication n'est identifiée au cours des 3 années suivantes.

**Tableau I:** Profil de sensibilité de la souche d’*Erysipelothrix rhusiopathiae* positif en hémoculture *Susceptibility profile of blood culture positive strain* Erysipelothrix rhusiopathiae

Famille d'antibiotiques	Nom de l'antibiotique	Résultat de l'antibiogramme
**β-lactamines: céphalosporines**	**Céfalotine**	**Sensible**
**Aminosides et Aminocyclitol**	**Gentamicine**	**Résistant**
**Macrolides**	**Erythromycine**	**Non testé**
	**Pristynamycine**	**Non testé**
**Quinolones**	**Ciprofloxacine**	**Sensible**
**Phénicolés**	**Chloramphénicol**	**Sensible**
**Tétracyclines**	**Tétracyclines**	**Sensible**
**Divers**	**Rifampicine**	**Résistant**
	**Vancomycine**	**Résistant**

## Discussion

Nous rapportons un cas d'endocardite infectieuse tricuspide à *E. rhusiopathiae* révélée par une sacro-iliite droite en Guyane. Si l'infection à *E. rhusiopathiae* est plutôt rare, elle n'a pratiquement jamais été rapportée dans la littérature indexée en Amérique latine. En effet, si l'on retrouve sur PubMed quelques publications, notamment brésiliennes sur cette infection chez l'animal [7, 10, 12], seuls 3 cas d'endocardite infectieuse ont été rapportés en Amérique latine à notre connaissance, 2 en Jamaïque en 1976 [17] et 1 en Argentine en 2002 [22].

À notre connaissance, seuls 23 cas d'infection ostéo-articulaire à *E. rhusiopathiae* ont été rapportés dans la littérature (Tableau II), et aucun cas de sacro-iliite n'a été décrit auparavant. La porte d'entrée est identifiée dans 17 cas. La période d'incubation varie de quelques semaines à plusieurs années. Dans 12 cas, des antécédents médicaux particuliers (immunosuppression, alcoolisme chronique, diabète) sont retrouvés, et dans 6 cas l'infection a lieu sur prothèse articulaire. Une bactériémie est associée dans 4 cas, et une endocardite infectieuse dans 3 cas.

**Tableau II:** Revue de la littérature des cas d'infections ostéo-articulaires à *Erysipelothrix rhusiopathiae* *Literature review of bone and joint infections caused by* Erysipelothrix rhusiopathiae

Référence	Âge/genre Profession ou activité Antécédent	Inoculation (si renseignée)	Type d'infection	Traitement
2017 États-Unis Alawdah [1]	12/H Allergie à la pénicilline	24 h auparavant Morsure de chien	Arthrite aiguë interphalangienne distale	Lavage chirurgical Lévofloxacine 4 semaines
2003 Grèce Allianatos *et al.* [2]	18/H	2 mois auparavant Plaie cutanée	Arthrite aiguë de genou	Lavage arthroscopique Pénicilline G et ciprofloxacine 5 semaines, puis clindamycine et ciprofloxacine 16 semaines
2012 Pologne Andrychowski *et al.* [3]	62/H Fermier Diabète	2 mois auparavant Plaie cutanée avec un os de porc	Spondylodiscite T5-T6 Empyème épidural	Drainage chirurgical de l'empyème Hémilaminectomie T5-T6 Céphalosporine (durée, relais non détaillé)
2018 France - Laon Belmenouar *et al.* [5]	51/H Cuisinier Éthylisme chronique, prolapsus mitral, cancer de prostate	Plaies cutanées répétées avec os, gibier	Spondylodiscite C5-C6 Suspicion d'endocardite infectieuse mitrale	Amoxicilline et lévofloxacine 4 mois
1996 Espagne Bianchi *et al.* [6]	55/H Boucher Hémodialyse	Plaie cutanée au travail	Arthrite aiguë d’épaule	Lavage chirurgical Pénicilline G 3 semaines
2015 France - Limoges Denes *et al.* [8]	57/H Fermier	19 ans plus tôt Encorné par une vache	Ostéoarthrite de la symphyse pubienne	Rifampicine et lévofloxacine 12 semaines
2000 États-Unis Dunbar et al. [9]	67/H Leucémie lymphoïde chronique, diabète		Arthrite aiguë du coude	Lavage arthroscopique
2018 France - Nancy Gazeau *et al.* [13]	82/M Prothèse de hanche bilatérale, prothèse de genou gauche		Infection chronique de prothèse de genou	Changement de prothèse en un temps Ceftriaxone 12 semaines Rechute un mois plus tard Changement de prothèse en deux temps Ceftriaxone et lévofloxacine 12 semaines
2019 Canada Groeschel *et al.* [15]	69/F Loisir: chasse Obésité, prothèse de genou	Consommation viande et poisson crus, chasse	Infection aiguë hématogène de prothèse de genou	Pénicilline G 6 semaines, puis amoxicilline 6 semaines
2010 France - Orléans Hocqueloux *et al.* [16]	68/F Éthylisme chronique, eczéma sous corticothérapie, prothèse de genou droite	Au cours du mois précédent En nourrissant un porc	Infection chronique de prothèse de genou	Changement de prothèse en deux temps Imipénem et ofloxacine 2 semaines, puis clindamycine et ofloxacine 24 semaines
2017 États-Unis Lorenz *et al.* [19]	48/H Loisir: pêche Matériel d'ostéosynthèse dans l’épaule droite, la hanche gauche, le genou droit Allergie à la pénicilline	2 semaines auparavant Abcès cutané secondaire à une plaie en pêchant	Spondylodiscite L5-S1 avec abcès prévertébrale et épidurite	Drainage chirurgical de l'abcès Ceftriaxone 8 semaines
2021 Irlande Mahon *et al.* [20]	65/H Jardinier Polyarthrite rhumatoïde sous méthotrexate et baracitinib, prothèse de hanche, obésité, tabagisme	Contact avec fumier de poule, dermohypodermite récidivante des mains (type rouget de porc)	Infection chronique de prothèse de hanche	Arrêt des traitements anti-inflammatoires Vancomycine puis benzylpénicilline/gentamycine/métronidazole puis amoxicilline/ciprofloxacine puis ciprofloxacine Changement de prothèse en deux temps prévu - seul un temps réalisé et ciprofloxacine suspensif
2021 France - Paris Maillard *et al.* [21]	51/H	1 an auparavant Plaie cutanée en coupant du poisson	Spondylodiscite L5-S1	Amoxicilline 2 semaines, puis ciprofloxacine 4 semaines
1998 Espagne Mera-Varela *et al.* [23]	36/H Pêcheur Éthylisme chronique		Arthrite aiguë du genou	Cloxacilline 2 semaines, puis érythromycine 4 semaines
2012 Inde Mukhopadhyay *et al.* [24]	5/H		Arthrite aiguë de hanche avec ostéomyélite fémorale proximale et myosite	Lavage arthroscopique Amoxicilline/clavunate 3 semaines
2008 Allemagne Neumann [25]	68/H Éthylisme chronique, cirrhose	Ulcère, contact avec des porcs	Arthrite aiguë de genou	Lavage arthroscopique Pénicilline G 3 semaines, puis amoxicilline/clavunate 3 semaines
2001 Canada Romney *et al.* [28]	67/F Éthylisme chronique, diabète Allergie à la pénicilline	Plaie en coupant du poisson	Suspicion d'ostéomyélite L3 Endocardite mitrale	Pénicilline G 6 semaines
2003 États-Unis Ruiz *et al.* [29]	76/H Fermier Loisir: pêche, jardinage Valve aortique mécanique		Arthrite chronique du genou gauche Épaississement de la valve mitrale d’étiologie indéterminée	Ponction articulaire répétée Pénicilline G 4 semaines
2007 Royaume-Uni Traer *et al.* [32]	76/H Tanneur Polyarthrite rhumatoïde, néphropathie lupique, corticothérapie systémique, prothèse de genou bilatérale	12 ans auparavant Plaie cutanée au travail	Infection chronique de prothèse de genou	Changement de prothèse en deux temps Pénicilline G et lévofloxacine 3 semaines puis clindamycine et lévofloxacine 7 semaines
2010 Danemark Troelsen *et al.* [33]	73/F Loisir: chasse Prothèse de hanche droite	Plaie chronique du talon	Infection chronique de prothèse de hanche	Changement de prothèse en deux temps Pénicilline G 3 semaines, puis amoxicilline 8 semaines
2014 Thaïlande Upapan *et al.* [34]	62/H Éthylisme chronique, cirrhose, diabète		Spondylodiscite L2-L3 avec abcès du psoas	Chirurgie (non précisée) Antibiothérapie (non précisée) 11 semaines
2019 Canada Wilson *et al.* [36]	71/H Pêcheur de crabe Insuffisance mitrale	Préparation du crabe au travail	Ostéomyélite L5 Bactériémie	Ceftriaxone 8 semaines
2003 Singapour Wong *et al.* [37]	41/F Lupus érythémateux disséminé avec corticothérapie systémique	Lavage hebdomadaire d'un aquarium	Arthrite chronique du genou	Lavage arthroscopique Pénicilline G 4 semaines, puis ciprofloxacine 2 semaines

La sacro-iliite est une infection rare, et représente 1 à 4% de l'ensemble des infections ostéo-articulaires [35]. Les symptômes sont la fièvre, une douleur inflammatoire locale, une impotence fonctionnelle. Le diagnostic est confirmé par l'imagerie et l'isolement d'un germe compatible. Les hémocultures sont positives dans 23 à 50% des cas [35], une ponction articulaire est nécessaire dans les cas incertains.

Les germes généralement impliqués dans les sacro-iliites sont: *Staphylococcus aureus* dans plus de la moitié des cas, puis les *Streptococci* sp. (en particulier *S. agalactiae* en période post-partum), puis les bactéries Gram négatif, *Mycobacterium tuberculosis* et *Brucella* sp. L'agent infectieux n'est pas retrouvé dans un tiers des cas. Quelques cas de sacro-iliite à germes inhabituels ont été rapportés: *Treponema pallidum, Neisseria gonorrhoeae, Neisseria meningitidis, Borrelia sp., Candida albicans, Pneumocystis jiroveci* ou *Cryptococcus neoformans* [35].

L'arthrite sacro-iliaque peut être favorisée par la grossesse et le post-partum. Pendant ces périodes, la mobilisation de l'articulation est facilitée par certaines hormones (relaxine). Nous avons initialement suspecté les 14 grossesses de la patiente comme facteur contributif d'une greffe infectieuse sur l'articulation sacro-iliaque. Cependant, l'espace interarticulaire sur le scanner ne semblait pas modifié, ce qui rend cette hypothèse peu probable. La patiente n'avait pas d'antécédents médicaux particuliers qui auraient pu faciliter le développement de l'infection.

*E. rhusiopathiae* a une forte capacité d'adhésion, due à ses protéines d'adhésion de surface RspA et RspB, partageant des homologies structurelles avec la Cna d'adhésion de *Staphylococcus aureus* [30]. En témoigne la fréquence de l'endocardite infectieuse en cas de bactériémie. Dans la revue de la littérature de Principe *et al.* (2016, 32 cas) une endocardite infectieuse était associée à 34% des bactériémies à *E. rhusiopathiae* [26]. Dans celle de Gorby et Peacock (1988, 49 cas), une endocardite infectieuse était associée dans 90% des cas [14].

## Conclusion

*E. rhusiopathiae* peut être responsable d'infections de localisation atypique. En témoigne ce cas clinique de sacro-iliite à *Erysipelothrix rhusiopathiae* associée à une endocardite tricuspide chez une femme sans facteur prédisposant. *E. rhusiopathiae* s'inscrit dans la liste des agents inhabituels responsables d'infections ostéoarticulaires, notamment en cas d'exposition professionnelle ou de contact animal.

## Liens D'intérêts

Les auteurs ne déclarent aucun lien d'intérêt.

## Contribution des Auteurs

Mathilde Kheng a rédigé le manuscrit. Loïc Epelboin a rédigé et corrigé le manuscrit. Jean Francky Alexis, Gaëlle Walter, Émilie Mosnier, Thomas Malmontet, Magalie Pierre Demar, Vilyn Tauch, Guillaume Vesin, Philippe Abboud, François Roque et Félix Djossou ont pris en charge la patiente et corrigé le manuscrit.
